# Supplementary material for: The impact of IOM recommendations on gestational weight gain among US women: An analysis of birth records during 2011–2019
Source: PLOS Glob Public Health. 2022 Jul 21;2(7):e0000815. doi: 10.1371/journal.pgph.0000815 (PMC10021552; doi:10.1371/journal.pgph.0000815)
Supplement: Attachment — Submitted filename: Response to reviewers May 31 2022.docx [file pgph.0000815.s001.docx]

May 31, 2022

Prof. Hanna Nalecz
Academic Editor
*PLOS Global Public Health.*

#### Dear Prof. Nalecz,

**PGPH-D-22-00245:** **The impact of IOM recommendations on gestational weight gain among US women: An analysis of birth records during 2011-2019.**

Thank you very much for reviewing above manuscript and inviting to submit a revision. The suggestion by you and the two anonymous referees are very helpful and would certainly make it a better paper. All your suggested revisions have been accommodated in this draft. The discussion was expanded adding a paragraph on the implications of the results and suggesting some recommendations that would improve the situation. The limitations are now in a separate paragraph. In addition to these suggested revisions there are a few minor editorial revisions too. A draft with all the changes to the original version marked-up has been submitted together with a clean version.

Thanking you,

Sincerely,

Vidhura Tennekoon

**Responses to reviewers:**

**PGPH-D-22-00245:** **The impact of IOM recommendations on gestational weight gain among US women: An analysis of birth records during 2011-2019.**

Thank you very much for reviewing above manuscript. Your suggestions are very helpful and would certainly make it a better paper. Your suggested revisions have been accommodated in this draft. The discussion was expanded adding the following paragraph:

*“While our results show a statistically significant effect of guidelines, that effect is far less than the intended effect. Addressing this gap requires clearly identifying why the gap exists. It has been suggested that this gap could be a result of limitations in the implementation procedure and if that is the case the appropriate solution is motivating healthcare providers through some action. However, knowledge about a guideline alone does not guarantee compliance, a best example is vaccine hesitancy. A woman’s prepregnancy BMI as well as the GWG is a result of her lifestyle choices though other factors such as genetics also play a role. The only tool available for physicians and other healthcare provides to implement IOM guidelines is patient education but lifestyle choices are hard to change through education alone and may require other types of interventions. A survey of pregnant women may help to understand the role of various factors causing the gap we identify here and respond as appropriate. Only through such a well-designed survey we can identify whether most women are unaware of the guidelines, they are aware but rely more on misinformation, or they simply ignore the guidelines. If most women are aware of the guidelines and they ignore those, a survey will also help to identify the reasons underlying such behavior.”*
